# Virulence and Antibiotic Resistance Profiles of *Proteus mirabilis* Strains Isolated From Broiler Chickens: Implications for Poultry and Public Health

**DOI:** 10.1002/vms3.70675

**Published:** 2025-11-19

**Authors:** Mohammad Reza Mohammadi, Niloofar Kiaheyrati, Mohadeseh Khakpour, Fatemeh Fardsanei, Fariba Najar Hoseini, Sona Sobhani, Mahsa Fallah Zavaraki, Gita Khorami Ejlali, Nazanin Alijani, Farhad Nikkhahi

**Affiliations:** ^1^ Medical Microbiology Research Center Qazvin University of Medical Sciences Qazvin Iran

**Keywords:** antimicrobial resistance, biofilm, broiler chickens, *Proteus mirabilis*, virulence gene

## Abstract

**Background:**

*Proteus mirabilis* is a common opportunistic zoonotic pathogen frequently linked to a wide range of human infections acquired in the community and hospital‐acquired infections. The emergence of antimicrobial resistance in *P. mirabilis*, largely driven by antibiotic overuse in both human and veterinary medicine, poses a growing global concern. This study aimed to investigate the association between biofilm formation, virulence gene expression and antibiotic resistance in *P. mirabilis* isolates collected from broiler chickens.

**Methods:**

A total of 50 isolates were confirmed as *P. mirabilis* from faecal and tissue samples of 100 broiler chickens by polymerase chain reaction (PCR). Antibacterial susceptibility was evaluated by the disc diffusion method, and nine virulence genes were screened by PCR. We also assessed the biofilm formation capability of the isolates using the crystal violet staining technique.

**Results:**

Among the 50 *P. mirabilis* isolates, the highest antimicrobial resistance was observed against chloramphenicol and trimethoprim/sulfamethoxazole (60%), whereas all isolates were fully susceptible to meropenem and cefepime. Multidrug resistance (MDR) was detected in 36% of isolates. Biofilm formation was demonstrated in 88% (*n* = 44) of isolates, with 74% exhibiting strong biofilm‐forming capacity. All isolates harboured *ireA, pmfA, atfA, zapA, hpmA* and *ptA* virulence genes, whereas *ucaA* was detected in 36% (*n* = 18) of isolates and *mrpA* was absent. The virulence gene *zapA* was significantly more prevalent in biofilm‐producing isolates compared to non‐biofilm producers (*p* ≤ 0.05).

**Conclusion:**

Poultry farms may represent an important reservoir of antimicrobial‐resistant *P. mirabilis*. Our findings underscore the need for responsible antimicrobial stewardship in veterinary medicine, particularly regarding agents critically important for human health, such as cephalosporins, fluoroquinolones, carbapenems.

## Graphical abstract

*Proteus mirabilis* strains isolated from broilers exhibited multidrug resistance, strong biofilm formation and diverse virulence genes, highlighting poultry as potential reservoirs of zoonotic pathogens and emphasizing the need for monitoring antimicrobial resistance and virulence in poultry production.

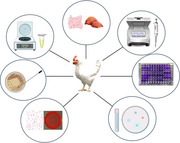

## 1 Introduction

Antimicrobial resistance (AMR) has emerged as one of the most urgent global public health threats, with increasing recognition of its impact on both human and veterinary medicine. A growing body of evidence indicates that food‐producing animals can act as important reservoirs of antimicrobial‐resistant microorganisms, thereby facilitating the transmission of resistance genes across the human–animal interface (Mehmood et al. 2023; Thabit et al. 2025). Prophylactic antibiotics are administered to healthy poultry to prevent infections, while feed preservatives maintain feed stability by inhibiting fungal and bacterial growth. In contrast to feed preservatives, the extensive non‐therapeutic use of antibiotics fosters the emergence and spread of antibiotic‐resistant bacteria (ARB), presenting considerable threats to both animal and human health (Nhung et al. 2016). Particularly, Enterobacterales strains of Gram‐negative bacteria (GNB) can acquire resistance by horizontal transfer of resistance genes via plasmids (Sasoon et al. 2025). The majority of the antibiotic‐resistant pathogens on the World Health Organization's (WHO) list are GNBs, which are a major concern for human health (Dandachi et al. 2018; Collignon et al. 2016).

*Proteus mirabilis* is a Gram‐negative bacillus primarily recognized for its swarming motility and urease activity. It is an opportunistic zoonotic pathogen that causes a broad spectrum of infections in humans, including urinary tract, wound, respiratory, ocular and gastrointestinal infections (Sanches et al. 2023). In poultry, *P. mirabilis* has the potential to infiltrate the intestinal tract of chickens and potentially contaminate poultry products through faecal contact during the slaughter process. In addition, post‐slaughter contamination can happen when meat products are processed, handled and stored. Such contamination pathways contribute to the dissemination of antimicrobial‐resistant bacteria along the food chain, thereby increasing the risk of human exposure (Barbour et al. 2012; Yu et al. 2021). Beyond its zoonotic potential, *P. mirabilis* is associated with cellulitis in broiler chickens, which not only affects flock health but also results in considerable economic losses through carcass condemnation during meat inspection (Sanches et al. 2020).

As previously stated, the excessive use of antibiotics has contributed to AMR in *P. mirabilis*, and this has become a growing global concern in recent years. This has caused substantial financial losses to animal husbandry and has hindered efforts to improve human health. Multidrug‐resistant (MDR) *P. mirabilis* poses a particular challenge for clinical treatment, as resistance to β‐lactams is mediated by β‐lactamases, including extended‐spectrum β‐lactamases (ESBLs), AmpC β‐lactamases and, less commonly, carbapenemases. Among these, ESBL‐producing isolates are especially concerning due to their association with elevated rates of morbidity and mortality, prolonged hospital stays and limited therapeutic options (Shaaban et al. 2022). The pathogenicity of *P. mirabilis* is primarily determined by its virulence factors, which enhance its ability to invade and persist within the host. These include the bacterial cell adhesion‐facilitating uroepithelial cell adhesin (UCA), the proteases *ptA* and *zapA*, which break down structural proteins and the immune system, respectively, and the haemolysins *hpmA* and *hlyA*, which are considered toxins to host cells and are involved in colonization. Cytotoxicity, adhesion capability and biofilm formation are critical during infection (Yu et al. 2021). Biofilm formation is a strategy that certain microbial species employ in order to endure harsh environmental conditions, as well as to improve their resistance to antibiotics and the immune system of the host (Yu et al. 2021; Sanches et al. 2020).

The present study aimed to characterize *P. mirabilis* strains isolated from broiler chickens, focusing on the prevalence of virulence genes as well as the phenotypic and genotypic patterns of antibiotic resistance. Given that poultry has been suggested as a potential reservoir for antimicrobial‐resistant bacteria with possible implications for human health, particularly in the context of One Health, this study addresses a gap in the limited data available from this region.

## 2 Materials and Methods

### 2.1 Bacterial Isolation and Identification

From August to December 2023, a total of 100 broiler chickens exhibiting clinical signs of cellulitis were selected for sampling. These broilers were identified by a veterinarian as affected and were removed from the production cycle. Tissue samples (liver and intestine) and faecal samples were collected directly from the cloaca for bacterial isolation and further analysis. All sampled chickens had routinely received enrofloxacin and colistin as part of farm management practices.

For the isolation of *P. mirabilis*, samples were incubated at room temperature (RT) in Tryptic Soy Broth (TSB) medium for 30 min. Subsequently, they were inoculated on Eosin Methylene Blue (EMB) agar plates and incubated overnight at 37°C. Cultures with one or two colonies were selected from each sample for bacterial identification. *P. mirabilis* isolates were identified using the API 20E biochemical identification system (bioMérieux, France) following the manufacturer's instructions. Further confirmation of *P. mirabilis* isolates was performed by polymerase chain reaction (PCR) for the *ureC* gene (Table 1). The purified isolates were preserved at −80°C in glycerol.

**TABLE 1:** Genes associated with virulence of *P. mirabilis* screened by PCR.

Genes	Primers sequences (5′➔3′)	Size (bp)	Ref.
*ureC*	F‐CCGGAACAGAAGTTGTCGCTGGA R‐GGGCTCTCCTACCGACTTGATC	510	Sanches et al. (2019)
*ireA*	F‐ACTACGATAACGAGCGCCAG R‐GCCCTAACTGGGGGAATACG	681	Sanches et al. (2019)
*mrpA*	F‐GAGCCATTCAATTAGGAATAATCCA R‐AGCTCTGTACTTCCTTGTACAGA	648	Rocha et al. (2007)
*ucaA*	F‐GCTTTTACATCCCCAGCGGT R‐GCTGCATTTGCTGGCTCATC	476	Sanches et al. (2019)
*pmfA*	F‐CAAATTAATCTAGAACCACTC R‐ATTATAGAGGATCCCTTGAAGGTA	617	Sanches et al. (2019)
*atfA*	F‐CATAATTTCTAGACCTGCCCTAGCA R‐CTGCTTGGATCCGTAATTTTTAACG	382	Zunino et al. (2000)
*ptA*	F‐CCACTGCGATTATCCGCTCT R‐ATCGGCAGAAGTGACAAGCA	686	Sanches et al. (2019)
*zapA*	F‐TATCGTCTCCTTCGCCTCCA R‐TGGCGCAAATACGACTACCA	332	Sanches et al. (2019)
*hpmA*	F‐GTTGAGGGGCGTTATCAAGAGTC R‐GATACTGTTTTGCCCTTTTGTGC	709	Cestari et al. (2013)

### 2.2 Antimicrobial Susceptibility Testing

Antibiotic susceptibility tests for *P. mirabilis* isolates were performed using the Kirby–Bauer disc diffusion method, according to the guidelines of the Clinical and Laboratory Standards Institute 2024 (CLSI). Cefotaxime (30 µg), clavulanic acid/cefotaxime (30 µg), ceftazidime (30 µg), ceftazidime/clavulanic acid (30 µg), cefoxitin (30 µg), cefepime (30 µg), meropenem (10 µg), levofloxacin (5 µg), gentamicin (10 µg), trimethoprim‐sulfamethoxazole (25 µg), chloramphenicol (30 µg) and norfloxacin (10 µg) were used in this study. *P. mirabili*s isolates were categorized as resistant, intermediate and susceptible based on the zone of inhibition in accordance with CLSI standards (CLSI, 2024). Isolates that displayed resistance to three or more antimicrobial classes were deemed MDR (Clinical and Laboratory Standards Institute 2024).

### 2.3 Phenotypic Detection of ESBL/AmpC‐Producing *P. mirabilis*

To identify these resistance phenotypes, the Combined Test method was used according to the CLSI guidelines 2024. Ceftazidime (30 µg) and ceftazidime/clavulanic acid, and cefotaxime (30 µg) and cefotaxime/clavulanic acid were administered. After preparing a suspension equivalent to 0.5 McFarland and culturing on Mueller–Hinton medium, cefotaxime/cefotaxime + clavulanic acid and ceftazidime + clavulanic acid discs were placed on Mueller–Hinton agar medium at an appropriate distance from each other, and the diameter of the no‐growth halo was measured after 18 h. If the halo of non‐growth increases by 5 mm or more in mixed discs containing clavulanic acid compared to the case without clavulanic acid, it means that this test is positive and confirms the production of ESBL (Rawat and Nair 2010). In addition, isolates resistant to cefoxitin and those exhibiting halo diameter growth inhibition of the cephalosporin disc with clavulanic acid, compared to the cephalosporin disc without clavulanic acid, which was less than 5 mm, were considered possible AmpC‐beta‐lactamase‐producing strains. *Klebsiella pneumoniae* standard isolate 700603ATCC was used as a positive control. Further phenotypic evaluation was performed using the AmpC induction test and boronic acid assay (Karapavlidou et al. 2005; Polsfuss et al. 2011).

### 2.4 Detection of β‐Lactamase‐Encoding Genes

Phenotypically confirmed ESBL‐, AmpC‐positive *Proteus* isolates underwent PCR using specific primers listed in Table 1 to detect ESBL genes (*bla*_TEM_, *bla*_SHV_ and *bla*_CTX‐M_), AmpC‐encoding genes (*bla*_ACC_, *bla*_CIT_, *bla*_EBC_, *bla*_FOX_, *bla*_MOX_ and *bla*_DHA_). The boiling method was used to extract genomic DNA. Briefly, the isolates were incubated at 37°C for 20 h after being cultured on trypticase soy agar (TSA). Two colonies were selected and inoculated into 400 µL of Tris‐EDTA (TE) buffer, heated at 100°C for 10 min, and then cooled down on ice for 15 min. Following centrifugation at 10,000 rpm, genomic DNA was collected from the supernatant and subjected to PCR for detection of AMR genes (Helmy and Wasfi 2014).

### 2.5 Biofilm Formation

Biofilm assays were performed in 96‐well polystyrene plates. We assessed the biofilm formation capability of the isolates by employing the crystal violet staining technique. This process was carried out in triplicate and repeated three times for each individual isolate. The verified strains of *P. mirabilis* were grown on TSA medium and incubated for 24 h at 37°C. Two to three colonies of a newly grown bacterial culture were cultivated in 5 mL of sterile TSB medium in a Falcon tube and placed in a shaker at 120 rpm. The culture was then incubated for 18–24 h at 37°C. The optical density (OD) of all the samples was measured using a spectrophotometer at a wavelength of 600 nm after a period of 18–24 h. A concentration of 0.5 McFarland, equivalent to 1–1.5 × 10^8^ CFU mL, was used in the test. In order to conduct the test, a strong biofilm‐producing strain of *Pseudomonas aeruginosa* was used as the positive control, and sterile TSB without bacteria served as the negative control, both inoculated into 96‐well flat‐bottom microplates and incubated at 37°C for 24 h. The media were subsequently removed and washed three times with phosphate buffered saline (PBS: pH 7.2). During the following step, the cells that produced biofilm were affixed to the plate using 95% methanol solution (200 µL/well) and fixed at RT for 15 min. Next, 200 µL of a 1% crystal violet solution was added into each well, and the plate was allowed to incubate at RT for 15 min to facilitate staining. After discarding the dye, the wells were washed thrice with PBS. To release the biofilm, 200 µL of 33% acetic acid was added to each well and the plate was shaken at RT for 15 min. The optical absorbance was quantified at a wavelength of 570 nm (OD570, ODC570) using a microtiter plate reader (BioTek, Epoch, USA). Biofilm formation was categorized into four groups based on OD measurements: negative (OD < ODc), weak (ODc < OD < 2 × ODc), moderate (2 × ODc < OD < 4 × ODc) and strong (OD > 4 × ODc), where ODc represents the OD of the negative control (Deilamani et al. 2023).

### 2.6 Detection of Virulence Genes

The virulence gene profiles of *P. mirabilis* isolates were determined using conventional PCR. Nine virulence genes were examined including *ireA* (siderophore receptors), *ptA* and *zapA* (proteases); *ucaA*, *pmfA*, *atfA* and *mrpA* (fimbriae); and *hlyA* and *hpmA* (haemolysins). The sequences and anticipated sizes of the amplified products are listed in Table 1. Bacterial DNA was obtained using the boiling extraction method (Sanches et al. 2020).

### 2.7 Statistical Analysis

SPSS software version 16 was used for conducting statistical analysis. The Kruskal–Wallis test was applied to compare the distribution of biofilm formation intensity among isolates obtained from different sample sources. Associations between individual virulence genes, bacterial sources, or antibiotic resistance patterns and biofilm formation strength were analysed using Chi‐square tests. The result was considered to have statistical significance if the *p*‐value was less than 0.05 (*p* < 0.05).

## 3 Results

### 3.1 Identification of *P. mirabilis*

Of the 100 isolates, 50 *P. mirabilis* isolates were identified by using the API 20E system (bioMérieux, France). Of these strains, 20 were isolated from broiler faeces, 16 from the liver and 14 from the intestine. A total of 50 isolates were found to be indole‐negative as well as positive on urease media, which produced a pinkish‐red colouration of the medium. Further confirmation was performed using PCR, and *ureC* was present in all 50 isolates (Table 2).

**TABLE 2:** Genotypic and phenotypic characteristics of virulence and antimicrobial resistance of *P. mirabilis* isolated from broilers.

Sample	Virulence genes	Biofilm	Phenotypic profile of resistance	Beta‐lactamase resistance genes
P1	*ureC, ireA, pmfA, atfA, ptA, zapA, hpmA*	Moderate	—	Not found
P2	*ureC, ireA, pmfA, atfA, ptA, zapA, hpmA*	Strong	NFX	Not found
P3	*ureC, ireA, ucaA, pmfA, atfA, ptA, zapA, hpmA*	Strong	—	Not found
P4	*ureC, ireA, ucaA, pmfA, atfA, ptA, zapA, hpmA*	Strong	C, SXT	Not found
P5	*ureC, ireA, pmfA, atfA, ptA, zapA, hpmA*	Strong	NFX	Not found
P6	*ureC, ireA, ucaA, pmfA, atfA, ptA, zapA, hpmA*	Strong	C	Not found
P7	*ureC, ireA, pmfA, atfA, ptA, zapA, hpmA*	Strong	NFX	Not found
P8	*ureC, ireA, pmfA, atfA, ptA, zapA, hpmA*	Strong	C, GM	Not found
P9	*ureC, ireA, pmfA, atfA, ptA, zapA, hpmA*	Strong	SXT, FOX	*bla*_DHA_
P10	*ureC, ireA, ucaA, pmfA, atfA, ptA, zapA, hpmA*	Strong	LEV, NFX, C, SXT	Not found
P11	*ureC, ireA, pmfA, atfA, ptA, zapA, hpmA*	Strong	C, SXT	Not found
P12	*ureC, ireA, pmfA, atfA, ptA, zapA, hpmA*	Strong	—	Not found
P13	*ureC, ireA, pmfA, atfA, ptA, zapA, hpmA*	Moderate	NFX, LEV, C, SXT, CAZ, CTX, FOX	*bla*_TEM_, *bla*_DHA_
P14	*ureC, ireA, pmfA, atfA, ptA, zapA, hpmA*	Weak	LEV, C, SXT, CTX	*bla*_TEM_, *bla*_DHA_
P15	*ureC, ireA, pmfA, atfA, ptA, zapA, hpmA*	Weak	LEV, C, SXT, CAZ, CTX, FOX	*bla*_TEM_, *bla*_DHA_
P16	*ureC, ireA, pmfA, atfA, ptA, zapA, hpmA*	Strong	NFX, LEV, C, SXT	Not found
P17	*ureC, ireA, ucaA, pmfA, atfA, ptA, zapA, hpmA*	Strong	LEV, C, SXT	Not found
P18	*ureC, ireA, pmfA, atfA, ptA, zapA, hpmA*	Strong	NFX, LEV, C, SXT	Not found
P19	*ureC, ireA, pmfA, atfA, ptA, zapA, hpmA*	Strong	LEV, C, SXT	Not found
P20	*ureC, ireA, ucaA, pmfA, atfA, ptA, zapA, hpmA*	Strong	C, SXT	Not found
P21	*ureC, ireA, pmfA, atfA, ptA, zapA, hpmA*	Strong	NFX, C, SXT	Not found
P22	*ureC, ireA, ucaA, pmfA, atfA, ptA, zapA, hpmA*	Strong	—	Not found
P23	*ureC, ireA, pmfA, atfA, ptA, zapA, hpmA*	Strong	NFX, SXT	Not found
P24	*ureC, ireA, ucaA, pmfA, atfA, ptA, zapA, hpmA*	Strong	—	Not found
P25	*ureC, ireA, ucaA, pmfA, atfA, ptA, zapA, hpmA*	Strong	—	Not found
P26	*ureC, ireA, ucaA, pmfA, atfA, ptA, zapA, hpmA*	Strong	NFX, C, SXT	Not found
P27	*ureC, ireA, pmfA, atfA, ptA, zapA, hpmA*	Strong	LEV, CTX, FOX	*bla*_TEM_, *bla*_DHA_
P28	*ureC, ireA, pmfA, atfA, ptA, zapA, hpmA*	Moderate	C, SXT, LEV	Not found
P29	*ureC, ireA, ucaA, pmfA, atfA, ptA, zapA, hpmA*	Strong	C, SXT, CTX, FOX	*bla*_TEM_, *bla*_DHA_
P30	*ureC, ireA, pmfA, atfA, ptA, zapA, hpmA*	None	C, LEV, CAZ, NFX	Not found
P31	*ureC, ireA, pmfA, atfA, ptA, zapA, hpmA*	Strong	C, SXT, LEV	Not found
P32	*ureC, ireA, pmfA, atfA, ptA, zapA, hpmA*	Strong	C, SXT, NFX	Not found
P33	*ureC, ireA, ucaA, pmfA, atfA, ptA, zapA, hpmA*	Strong	SXT, LEV, CAZ, CTX, FOX	*bla*_TEM_, *bla*_DHA_
P34	*ureC, ireA, ucaA, pmfA, atfA, ptA, zapA, hpmA*	Strong	C, NFX	Not found
P35	*ureC, ireA, pmfA, atfA, ptA, hpmA*	Strong	C, SXT	Not found
P36	*ureC, ireA, pmfA, atfA, ptA, zapA, hpmA*	Strong	SXT, LEV	Not found
P37	*ureC, ireA, pmfA, atfA, ptA, hpmA*	None	SXT, LEV	Not found
P38	*ureC, ireA, pmfA, atfA, ptA, hpmA*	None	SXT, LEV	Not found
P39	*ureC, ireA, pmfA, atfA, ptA, zapA, hpmA*	Strong	C, SXT, NFX	Not found
P40	*ureC, ireA, ucaA, pmfA, atfA, ptA, zapA, hpmA*	Strong	C, SXT	Not found
P41	*ureC, ireA, pmfA, atfA, ptA, zapA, hpmA*	None	C, LEV	Not found
P42	*ureC, ireA, pmfA, atfA, ptA, zapA, hpmA*	None	C, SXT	Not found
P43	*ureC, ireA, ucaA, pmfA, atfA, ptA, hpmA*	Strong	LEV, NFX	Not found
P44	*ureC, ireA, pmfA, atfA, ptA, zapA, hpmA*	Strong	SXT, NFX	Not found
P45	*ureC, ireA, ucaA, pmfA, atfA, ptA, zapA, hpmA*	Strong	C, SXT	Not found
P46	*ureC, ireA, pmfA, atfA, ptA, zapA, hpmA*	None	—	Not found
P47	*ureC, ireA, ucaA, pmfA, atfA, ptA, zapA, hpmA*	Strong	C, SXT, NFX	Not found
P48	*ureC, ireA, ucaA, pmfA, atfA, ptA, zapA, hpmA*	Strong	C, NFX	Not found
P49	*ureC, ireA, pmfA, atfA, ptA, hpmA*	Moderate	C	Not found
P50	*ureC, ireA, pmfA, atfA, ptA, zapA, hpmA*	Weak	—	Not found

### 3.2 Antimicrobial Susceptibility Phenotypic Profile

**FIGURE 1:**
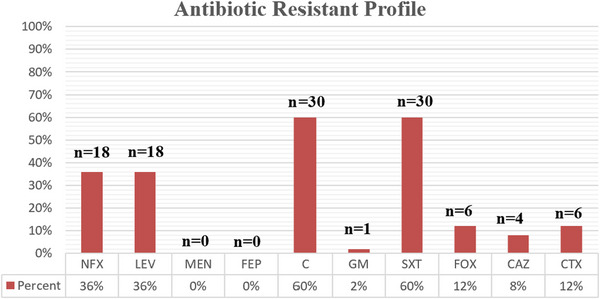
Antibiotic susceptibility profile of 50 *Proteus mirabilis* isolates.

The resistance patterns of the tested 5 antimicrobial categories, which consist of 14 antimicrobial agents, against all *P. mirabilis*, are detailed in Figure 1. *P. mirabilis* exhibited the highest antibiotic resistance rate to chloramphenicol and trimethoprim/sulfamethoxazole 60% (30/50). As shown in Figure 1, of 50 *P. mirabilis* isolates examined, 18 (36%), 18 (36%), 6 (12%), 4 (8%) and 6 (12%) were resistant to norfloxacin, levofloxacin, cefotaxime, ceftazidime and cefoxitin, respectively. Regarding the ceftazidime and cefotaxime used in our study, 4 (8%) and 0 (0%) of isolates showed intermediate resistance, respectively. In addition, the highest sensitivity was observed for meropenem and cefepime as 100% (50/50), and gentamicin as 98% (49/50). Based on the present findings, 18 (36%) isolates were MDR. Isolates were considered MDR exhibiting resistance to three or more classes of antimicrobials.

### 3.3 Phenotypic Detection of ESBL/AmpC‐Producing *P. mirabilis*

A combined disc assay was used to check for the potential presence of ESBLs in all ceftazidime‐ or cefotaxime‐resistant isolates. Of 50 *P. mirabilis* isolates, 24% (*n* = 12) of isolates were β‐lactamase producers. Six isolates were ESBL producers, as determined by the Combined Disk Test (CDT). Also, isolates resistant to cefoxitin were selected for AmpC production. Among these isolates, six were AmpC producers. Isolates suspected of AmpC production were further confirmed using the phenylboronic acid (PBA) test.

### 3.4 Detection of β‐Lactamase‐Encoding Genes

Among the 50 isolates included in this study, in relation to the ESBL and AmpC genes responsible for conferring resistance to β‐lactam antimicrobials, 100% (6/6) of isolates were positive for AmpC genes for *bla_DHA_* gene, and 100% (6/6) of isolates were positive for ESBL for *bla*_TEM_ (Table 2).

### 3.5 Biofilm Formation

Our findings demonstrated a capacity for biofilm formation in polystyrene plates, in which 37 samples strongly, 4 isolates moderately, 3 isolates weakly formed biofilm. The results are shown in Table 2.

### 3.6 Detection of Virulence Genes

**FIGURE 2:**
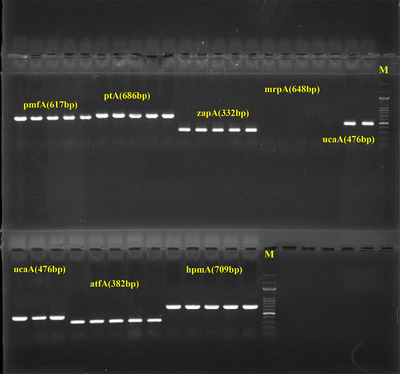
Gel electrophoresis of PCR products of virulence‐associated genes in Proteus mirabilis.

**FIGURE 3:**
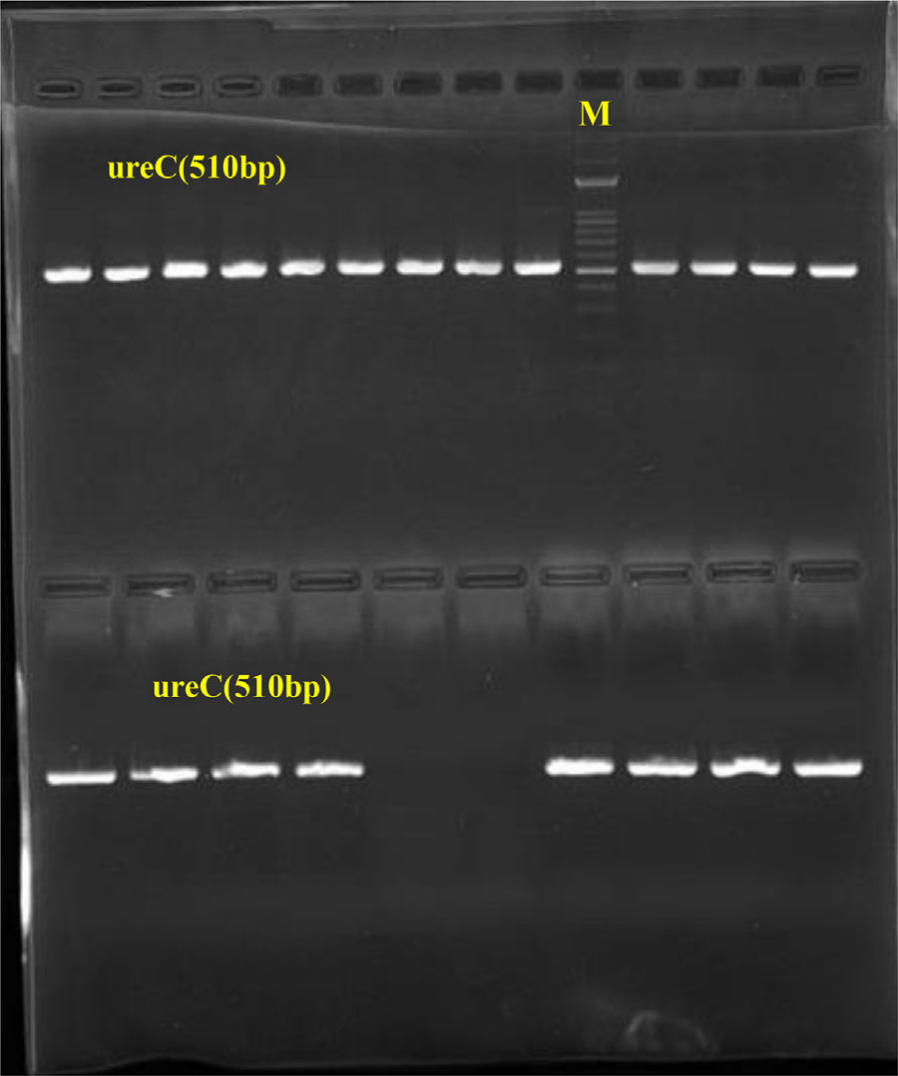
Gel electrophoresis of PCR amplification of the ureC gene in Proteus mirabilis.

The studied bacterial isolates contained a wide range of virulence genes that enabled and contributed to the progression of infection in humans. All isolates carried *ireA, pmfA, atfA, zapA, hpmA* and *ptA* genes (Table 2). Of the other virulence genes, *ucaA* was found in 18 (36%) isolates, whereas *mrpA* was not detected in any isolate. The electrophoretic profiles of PCR‐amplified virulence genes are presented in Figures 2 and 3.

## 4 Discussion

In this study, we characterized *P. mirabilis* strains isolated from broiler chickens, focusing on virulence factors, biofilm formation and AMR patterns. The isolation of 50 *P. mirabilis* strains from infected broilers in this study highlights the potential role of poultry as reservoirs for antimicrobial‐resistant pathogens. The poultry farm in our study followed standard biosecurity measures, including regular cleaning of housing, controlled access for personnel and vaccination programs. In poultry, *P. mirabilis* colonization is typically acquired via horizontal transmission through contaminated litter, water, feed, or contact with other birds. Such differences in farm management and hygiene practices may contribute to the observed variation in AMR and biofilm formation among *P. mirabilis* isolates.

Antimicrobial susceptibility tests revealed high resistance to most antimicrobials typically employed in the treatment of infections caused by Enterobacteriaceae. This is consistent with reports of increasing *P. mirabilis* drug resistance in recent years, which has made treatment of associated infections progressively more challenging (Chakkour et al. 2024). Our findings indicate a statistically significant overall disparity in the prevalence of antibiotic resistance among sample sources (Kruskal–Wallis, *p* = 0.0416). Mean ranks indicate a higher resistance burden in the intestine and faeces compared with the liver. Biologically, this pattern is plausible because the intestine and faeces are dense and diverse microbial communities that are frequently exposed to orally administered antimicrobials and environmental residues, resulting in increased selective pressure and horizontal gene transfer. Conversely, isolates recovered from internal organs, such as the liver, likely reflect a subset of bacteria associated with invasive infection and may have a different ecological and selective background. The extensive use of antimicrobials in these farms likely exerts strong selection pressure, contributing to the emergence and dissemination of drug‐resistant strains. This is largely attributed to the routine use of antimicrobial agents in veterinary medicine for prophylaxis, growth promotion and therapeutic purposes (Lordejani et al. 2025). However, the lack of genomic analysis in this study limits our ability to determine whether the observed resistance is mediated by mobile genetic elements or clonal expansion, highlighting the need for future studies to include whole genome sequencing and plasmid profiling.

It is important to emphasize the significant rate of resistance to chloramphenicol and trimethoprim/sulfamethoxazole (60%), which was similar to those isolated from chicken products in China by Wong et al. (2013) and Sun et al. (2020). Although resistance to fluoroquinolones was observed, the prevalence in our isolates was lower than in previous studies, where Kwiecińska‐Piróg et al. (2013) reported 40% and Ramatla et al. (2024) reported 62%. In our study, no resistance was observed to carbapenems (meropenem) or to the fourth‐generation cephalosporin cefepime (0%). This finding is encouraging and suggests that these last‐resort antimicrobials are still effective against *P. mirabilis* in poultry in our setting. It is interesting to compare our findings with those from Yen et al. in the Mekong Delta, Vietnam. In their study, 2.1% of poultry had CRE/CRAB carriage (Yen et al. 2022). In contrast, Ramatla et al. (2024) found that *P. mirabilis* isolated from broilers in South Africa had low but detectable resistance rates of 8% for both imipenem and ertapenem. Although no carbapenem resistance was detected in our isolates, occasional reports from poultry in other countries suggest that such findings should be regarded with caution, since resistance incidence varies greatly by geography and detection method.

ESBL genes were detected in six (12%) *P. mirabilis* isolates, a prevalence lower than that reported from Bangladesh by Mishu et al. (2022). Notably, all ESBL‐producing isolates (100%) were biofilm producers, although not all biofilm‐producing isolates harboured ESBL genes, a difference that was statistically significant. While ESBL‐producing isolates may have zoonotic potential, direct transmission from chickens to humans was not assessed in this study and should be considered a limitation. In addition, six isolates carried AmpC genes; in contrast, a previous study from Egypt reported only a single *P. mirabilis* isolate harbouring AmpC (Shaaban et al. 2022).

In our study, 36% of *P. mirabilis* isolates were identified as MDR, a prevalence lower than the 46% reported from poultry isolates in China (Sun et al. 2020). Differences in antimicrobial usage policies, farm management, sampling strategies, geographic properties, sample types and sample sizes can lead to variations. Although there is an ongoing debate on the transfer of MDR organisms from animals to humans, several studies have demonstrated a direct connection between direct contact with farm animals and the acquisition of bacterial resistance (Abou‐Jaoudeh et al. 2024). Therefore, implementing appropriate measures to mitigate the spread of AMR in animal farming is crucial to protect both animal and human health. In addition, *P. mirabilis*, which is derived from animals, is a zoonotic pathogen that possesses a variety of virulence genes. The isolates in this study carried virulence genes, such as *ireA* (siderophore receptor), *mrpA*, *ucaA*, *pmfA*, *atfA*, *ptA* (proteases), *zapA* and *hpmA* (haemolysin) genes. Most of them had the highest detection rate of 100%, and the lowest was *mrpA* at 0%, which is similar to study conducted by Ramatla et al. (2024). In relation to the genes coding haemolysins, all isolates were found to possess the *hpmA* gene, whereas *hlyA* was not detected in any of these. These results are consistent with recent research indicating a higher prevalence of *hpmA* compared to *hlyA* in *P. mirabilis*. (Cestari et al. 2013; Swihart and Welch 1990). Our results are particularly alarming, as all *P. mirabilis* strains isolated from chickens, produced a strong or very strong biofilm. Biofilm formation in the host serves as a protective mechanism for bacteria against the immune system and antimicrobial agents, while also playing a role in the prolonged existence of infection (Sun et al. 2020). Our analysis revealed a significant association between sample source and biofilm intensity (*p* = 0.018). Strong biofilm formation was notably more prevalent among faecal (85.0%) and liver isolates (81.3%) compared with intestinal isolates (50.0%). This may indicate variations in selective pressures and ecological niches: faecal isolates encounter dense microbial communities and remnants of orally‐administered antimicrobials, which may promote biofilm‐forming and horizontally‐transmissible strains; liver isolates presumably originate from subpopulations adapted to persistent or invasive phenotypes (İnce and Akan 2024).

Among the examined genes, only *zapA* exhibited a statistically significant association with biofilm production (*p* = 0.038), indicating that its presence may contribute to stronger biofilm formation. Other genes, including *ureC, ireA, mrpA, ucaA, pmfA, atfA, ptA* and *hpmA*, showed no significant correlation with biofilm intensity (*p* > 0.05). Similar patterns were reported in studies by Sun et al. (2020) and Mishu et al. (2022), where biofilm‐producing isolates showed a higher prevalence of virulence genes than non‐producers. These findings highlight the need for responsible antimicrobial use in animal farming, under strict veterinary supervision, and for exploring alternative disease control strategies. Implementing monitoring and surveillance systems is also essential to track the prevalence and spread of AMR in animal populations.

While this study provides valuable data on AMR, biofilm formation and virulence gene distribution in *P. mirabilis* from poultry, several limitations should be considered. The relatively small sample sizes for different source subgroups may limit statistical power, and the absence of genomic analyses restricts insights into the potential role of mobile genetic elements. Moreover, although poultry may serve as a reservoir for antimicrobial‐resistant bacteria, the implications for human colonization and zoonotic transmission remain tentative. Future research incorporating whole genome sequencing and plasmid profiling could further elucidate the mechanisms underlying resistance gene mobility and potential public health risks.

## 5 Conclusion

In conclusion*, P. mirabilis* strains found in broiler chickens exhibit a wide range of virulence factors and are resistant to a number of antibiotics commonly used to treat infections in humans. The high occurrence on broilers suggests a potential risk for human colonization and infection. To reduce the risk of AMR, it is recommended to prioritize veterinary‐approved antibiotics and follow prudent use protocols, rather than routinely employing drugs critical for human medicine. These findings also highlight the potential value of integrated One Health strategies, which consider the interconnections between human, animal and environmental health, in mitigating AMR in food‐producing animals.

## Author Contributions

**Mohammad Reza Mohammadi**: conceptualization, data curation, investigation, methodology, resources, visualization, writing – original draft. **Niloofar Kiaheyrati**: conceptualization, data curation, investigation, methodology, resources, visualization, writing – original draft. **Mohadeseh Khakpour**: methodology, writing – review and editing. **Fatemeh Fardsanei**: data curation, methodology, writing – review and editing. **Fariba Najar Hoseini**: methodology. **Sona Sobhani**: methodology. **Mahsa Fallah Zavaraki**: methodology. **Gita Khorami Ejlali**: writing – review and editing. **Nazanin Alijani**: writing – review and editing. **Farhad Nikkhahi**: conceptualization, funding acquisition, supervision, writing – original draft, writing – review and editing.

## Funding

The authors have nothing to report.

## Ethics Statement

All methods in the current study were performed in accordance with the guidelines of the Ethics Committee of Qazvin University of Medical Sciences with approval number IR.QUMS.REC.1402.188.

## Conflicts of Interest

The authors declare no conflicts of interest.

## Data Availability

The data that support the findings of this study are available from the corresponding author upon reasonable request.
